# Delayed Labor and Death of Patient

**Published:** 1877-03-20

**Authors:** 


### DELAYED LABOR, AND DEATH OF PATIENT.

In the *Detroit Medical Review* Dr. Stoddart reports a case of delayed labor. He was called in consultation to see Mrs. W.

She had become pregnant about a year previously and had expected to be confined about twelve weeks previous to my call. At the expected time labor pains came on feebly. She sent for the family physician. He judged from the character of the pains that she would not yet be confined, so after waiting some little time he administered an opiate. This had the effect to soon quiet all pain. Her physician left her, giving orders that he was to be called if labor came on again. He did not hear anything more from his case for a long time, when he was again called. He found the pains even more inefficient than before. After waiting some time, and finding that the pains were again dying away, the medical attendant gave ergot in increasing doses. This had no effect whatever, except it might be to quiet all labor pains. Till now the motion of the fœtus was quite vigorous, but soon the motions became more and more feeble, till they were felt no more. About a week or ten days after the ergot was administered she was taken with a chill. This was probably caused by the death of the child, and the reflex influence of it upon her; at least, from that time no more motion was felt. Nothing had heretofore been done, except the administration of the ergot, to induce labor. The physician believing, as was now doubtless the case, that the fœtus was dead, thought labor would now surely come on, and waited patiently from day to day, and even from week to week, for its event. But it did not come on. Counsel was called in, but for some unknown reason no surgical interference was recommended to help the patient to cast off her now dead and decaying child. In some partial explanation of this failure to relieve her, it was said that the patient was excessively tender, and that even to make a digital examination gave her the most excruciating pain. At one time the physician introduced a little way into the uterus a flexible catheter, and succeeded in bringing away a small quantity of fluid. But this was all that was done for her. No doubt this was a grave and fatal mistake. From day to day the waters dribbled away, then a sort of purulent and offensive discharge came on, and yet no help was offered the patient to enable her to cast off her burden. Days and weeks thus passed away, and counsel was again called from a distance; but it was decided that the time for successful surgical interference was past; that adhesion had taken place between the walls of the abdomen and the uterus, and that suppuration had already occurred, and that pus would soon find its way to the surface. A few days more proved the prognosis to be only too true, for two or three abscesses rapidly formed on the abdomen. These, when opened, discharged large quantities of pus, which was soon followed by a sanious and most disgusting fluid. This was kept up from week to week till her death. Other abscesses and fistulous openings made their appearance, and at the time I saw her there were six or seven of them.

Of course I could advise nothing. I probed one or two of the older openings, and thought that I felt bone at the bottom of one. On the first day of July she died, a little over thirteen months from the time she became pregnant. I was called upon to aid in the *post mortem* examination. The attending physician maintained that it was a case of extra-uterine pregnation. After cutting through the partially ulcerated walls of the abdomen, the uterus was found adherent on the whole of its anterior side to the abdominal parieties. The uterus was filled with the partially decayed remains of a full-grown fœtus. The ovaries and fallopian tubes were normal and undisturbed. The os uteri was slightly occluded by adhesive inflammation, and not dilated in the least. Very slight force of the finger broke up the adhesion of the os, so this could have been no bar to natural labor. The patient died solely from exhaustion and septic poisoning, derived from the decaying fœtus. This was a case, probably, of paralysis of the muscular structure of the uterus, whether functional or otherwise could not be determined. The examination clearly taught that surgical measures should have been employed at least after the death of the child in utero. A life, and perhaps two, were sacrificed to the inertia of the physician. The os uteri ought to have been promptly dilated, and the fœtus removed at all hazards. If it could have been done before the death of the child, perhaps it would have excited the functionally paralyzed uterus to labor, and thus both lives have been spared.—*Canada Lancet.*

